# A Water-Soluble Polysaccharide from the Fruit Bodies of *Bulgaria inquinans* (Fries) and Its Anti-Malarial Activity

**DOI:** 10.1093/ecam/neq070

**Published:** 2011-05-03

**Authors:** Hongtao Bi, Han Han, Zonghong Li, Weihua Ni, Yan Chen, Jingjing Zhu, Tingting Gao, Miao Hao, Yifa Zhou

**Affiliations:** ^1^School of Life Sciences, Northeast Normal University, Changchun 130024, China; ^2^Baicheng Medical College, Baicheng 137000, China

## Abstract

A water-soluble polysaccharide (BIWS-4b) was purified from the fruit bodies of *Bulgaria inquinans* (Fries). It is composed of mannose (27.2%), glucose (15.5%) and galactose (57.3%). Its molecular weight was estimated to be 7.4 kDa (polydispersity index, Mw/Mn: 1.35). Structural analyses indicated that BIWS-4b mainly contains (1 → 6)-linked, (1 → 5)-linked and (1 → 5,6)-linked *β*-Gal*f* units; (1 → 4)-linked and non-reducing terminal *β*-Glc*p* units; and (1 → 2)-linked, (1 → 6)-linked, (1 → 2,6)-linked and non-reducing terminal *α*-Man*p* units. When examined by the 4-day method and in a prophylactic assay in mice, BIWS-4b exhibited markedly suppressive activity against malaria while enhancing the activity of artesunate. Immunological tests indicated that BIWS-4b significantly enhanced macrophage phagocytosis and splenic lymphocyte proliferation in malaria-bearing mice and normal mice. The anti-malarial activity of BIWS-4b might be intermediated by enhancing immune competence and restoring artesunate-suppressed immune function. Thus, BIWS-4b is a potential adjuvant of anti-malaria drugs.

## 1. Introduction

Many fungi are used as traditional medicines in the treatment of various human diseases such as hepatitis, hypertension, hypercholesterolemia and gastric cancer [1–4]. Recently, fungal polysaccharides have received considerable attention as an important class of bioactive substances because of their potent biological and pharmacological activities, especially immunological and antitumor activities [5–8]. Discovery and evaluation of new bioactive polysaccharides from medicinal fungi has emerged as one of the hot research fields in chemistry and biology. Due to their immunological activities, some fungal polysaccharides could protect hosts from microbes and parasites such as malaria [9].

The non-lichenized ascomycete *Bulgaria inquinans* (Fries) is an edible wood-inhabiting ascomycete that grows on freshly felled oak and commonly found in the Changbai Mountain area of China. It has been used as food and folk antitumor medicine for a long time. Several small molecule compounds isolated from the fruit bodies of *B. inquinans* (Fries) have been found to have antitumor [10], antipruritic and antierythema activities [11]. However, the polysaccharides from *B. inquinans* (Fries) have not been thoroughly studied. In a previous study, we fractionated polysaccharides from *B. inquinans* (Fries) and a low molecular weight *β*-(1→6)-d-glucan was characterized [12]. In the present article, we describe the characterization of the more complex heteropolysaccharide fraction BIWS-4b from *B. inquinans* (Fries) and its anti-malarial activity *in vivo* as well as a possible mechanism of action.

## 2. Methods

### 2.1. Materials and Chemicals

Fruit bodies of *B. inquinans* (Fries) were collected from Changbai Mountain, China and identified by Professor Hongxing Xiao at Northeast Normal University in Changchun, China. A voucher specimen (No. 20070802) was deposited at the School of Life Sciences, Northeast Normal University.

Artesunate was purchased from Guilin Pharmaceutical Co., Ltd., China. 3-(4,5-Dimethylthiazol-2-yl)-2,5-diphenyltetrazolium bromide (MTT), concanavalin A (ConA), lipopolysaccharide (LPS), Sepharose CL-6B and Sephadex G-75 were purchased from Sigma Chemical Co. RPMI-1640 medium was obtained from Gibco Invitrogen Co. The complete RMPI-1640 medium used for immunological testing had a pH of 7.4 and was supplemented with penicillin (100 IU mL^−1^), streptomycin (100 *μ*g ml^−1^) and 10% fetal bovine serum (FBS). All other reagents were of analytical grade and made in China.

### 2.2. Animals

An equal number of male and female ICR mice (Grade II, 6–8 weeks old), weighing 18–22 g, were purchased from the Pharmacology Experimental Center of Jilin University (approval number: SCXK (Jilin) 2002-0002, Changchun, China) and acclimatized for one week prior to use. All mice were housed under standard conditions at 24 ± 1°C with humidity of 50 ± 10% and a 12/12 h light/dark cycle. Rodent laboratory chow pellets and tap water were supplied *ad libitum*. All procedures were in strict accordance with PR China legislation and with the guidelines established by the School of Life Sciences of Northeast Normal University regarding the use and care of laboratory animals. The protocol was approved by the University's Committee for Animal Experiments and Science & Technology Department of Jilin Province (approval number: SCXK (Jilin) 2003-0001).

### 2.3. Isolation and Purification of BIWS-4b

**Figure 1:**
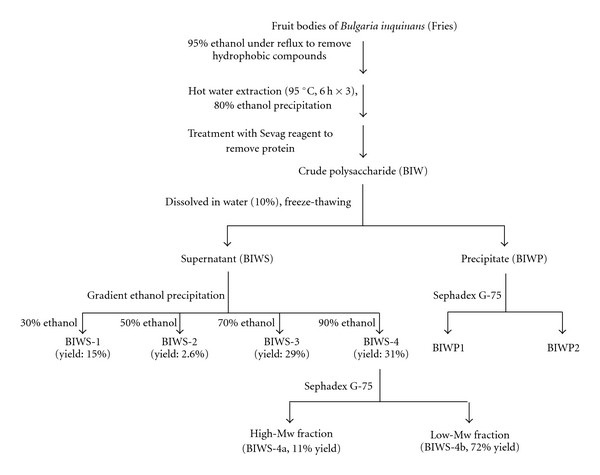
Extraction and purification procedure of BIWS-4b.

The isolation of polysaccharides from fruit bodies of *B. inquinans* (Fries) was carried out as previously described by our group [12]. In brief, dried fruit bodies of *B. inquinans* (Fries) were extracted with 95% ethanol under reflux for 12 h to remove hydrophobic compounds and then extracted three times with hot distilled water (6 h for each extraction, 90–95°C, 1 : 20 w/v). The extracts were combined and concentrated to one-tenth of the original volume, and then ethanol was added to the concentrated extracts up to 80% to precipitate the crude polysaccharides. Crude polysaccharides were collected by centrifugation, and then treated with Sevag reagent [13] to remove free proteins to give the polysaccharide fraction (BIW). The aqueous solution of BIW (10% w/v) was frozen at –20°C and then allowed to thaw slowly at 4°C, which yielded the soluble fraction (BIWS) and insoluble fraction (BIWP). BIWP was fractionated and characterized in our previous study [12]. In the present study, BIWS was dissolved in water (10% w/v), and then fractionated by gradient precipitation in 30, 50, 70 and 90% ethanol resulting in four fractions BIWS-1, BIWS-2, BIWS-3 and BIWS-4, respectively. BIWS-4 was further fractionated on a column (100 × 1.5 cm) of Sephadex G-75 and eluted with 0.15 M NaCl to give two fractions: BIWS-4a collected in the void volume and BIWS-4b collected as a main portion. After dialysis in tubing (Mw cut-off 3.5 × 10^3^ Da for global protein), the fractions were concentrated and lyophilized to give the pure polysaccharide fraction BIWS-4b. The extraction and purification procedure was shown in Figure 1. All gel filtration chromatographies were monitored by assaying carbohydrate content. 

### 2.4. Analytical Methods

The total carbohydrate content was determined by the phenol-H_2_SO_4_ method using glucose as a standard [14]. Protein content was determined in a Bradford assay using bovine serum albumin as the standard [15]. Contaminant endotoxin was analyzed in a limulus amebocyte lysate (LAL) assay using an E-TOXATE kit (Sigma, St. Louis, USA) according to the manufacturer's instructions.

Monosaccharide analysis was performed as described by Honda et al. [16]. Briefly, sample (2 mg) was hydrolyzed with 2 M CF_3_COOH (1.0 ml) at 120°C for 3 h. The hydrolyzed-products (monosaccharides) were derivatized with 0.5 M 1-phenyl-3-methyl-5- pyrazolone (PMP) and 0.3 M NaOH. After neutralization with 0.3 M HCl, the PMP-derivatives were analyzed in a Shimadzu 10Avp HPLC system equipped with a Shim-pak VP-ODS column (150  ×  4.6 mm i.d.) with guard column and monitored by UV absorbance at 245 nm.

The specific rotation was measured at 20 ± 1°C with an automatic polarimeter (Model WZZ-2B, China). UV-Vis absorbance spectra were recorded with a UV-Vis spectrophotometer (Model SP-754, China). FT-IR spectra were obtained on a Nicolet 560 FT-IR spectrometer with DTGS detector in a range of 400–4000 cm^−1^. The samples were ground with KBr powder and then pressed into 1 mm pellets for FT-IR measurements.

The ^13^C NMR spectrum was recorded using a Bruker 5 mm Broadband Observe probe at 20°C in a Bruker Avance 600 MHz spectrometer (Germany) operating at 150 MHz. Sample (20 mg) was dissolved in D_2_O (99.8%, 0.5 ml), freeze-dried, redissolved in D_2_O (0.5 ml) and centrifuged to remove excess sample. The experiment was recorded using standard Bruker software.

### 2.5. Homogeneity and Molecular Weight Determination

Homogeneity and molecular weight were determined in a Shimadzu 10Avp HPLC system equipped with a 10Avp Pump and RID-10A Refractive Index Detector. The system was linked to the gel filtration column TSK-G3000 PW_XL_ and sample was eluted with 0.2 M NaCl at a flow rate of 0.55 ml min^−1^ at 35.0 ± 0.1°C. The column was calibrated by standard dextrans (50, 25, 12, 5 and 1 kDa) using linear regression. Sample concentration upon injection was 5 mg ml^−1^ and approximately 20 *μ*l was injected.

### 2.6. Methylation Analysis

The methylation analysis was carried out according to the method of Needs and Selvendran [17]. In brief, BIWS-4b (10 mg) was dissolved in DMSO (1.5 ml) and then methylated by treatment with a NaOH/DMSO suspension (1 ml) and iodomethane (1 ml). The methylated polysaccharide was extracted with CHCl_3_ and then the solvent was removed by vacuum evaporation. The progress of methylation was followed by the disappearance of the –OH band (3200–3400 cm^−1^) in the FT-IR spectrum. The per-*O*-methylated polysaccharides were hydrolyzed subsequently by HCOOH (85%, 0.5 ml) for 4 h and CF_3_COOH (2 M, 1 ml) for 6 h at 100°C. Partially methylated sugars in the hydrolysate were reduced by NaBH_4_ and then acetylated with pyridine (0.5 ml) and acetic anhydride (0.5 ml) at 90°C for 1 h. The resulting mixture of alditol acetates was analyzed by GC-MS. GC-MS was carried out on an Agilent 6890N-5975 instrument equipped with a HP-5 column.

### 2.7. Preliminary Anti-Malaria Testing In Vivo

#### 2.7.1. Parasite Inoculation

The *Plasmodium yoelii* strain 17XL was a gift from Prof. Cao Yaming (Department of Immunology, China Medical University) and blood stage parasites were stored in liquid nitrogen. The standard inoculum consisted of 5 ×10^7^ ml^−1^ *P. yoelii* parasitized erythrocytes from a donor mouse, which was used to infect mice intraperitoneally.

#### 2.7.2. Suppressive Activity on Early Infection (4-Day Test)

Suppression of early infection was evaluated using a 4-day test described by Peters and Robinson [18]. Briefly, after inoculating with 0.2 ml of standard inoculum, the mice were randomly divided into six groups of six mice each. The polysaccharide-treated groups were given BIWS-4b of different dosages (50, 100 and 200 mg kg^−1^), and the co-treated group was given the mixture of BIWS-4b (100 mg kg^−1^) and artesunate (14 mg kg^−1^). A negative control group was administered physiological saline and positive control groups were treated with artesunate at doses of 14 or 28 mg kg^−1^. The drugs were orally administered to mice in 0.2 ml doses daily for 4 consecutive days (Days 0–3). On Day 4, blood smears were generated from tail blood and stained with Giemsa stain. Drug activity was assessed by evaluating the smears under a microscope. Parasitemia (%) was calculated by dividing the number of parasitized erythrocytes by the total number of erythrocytes. Average chemosuppression (%) was calculated as 100  ×  [(*A* – *B*)/*A*], where *A* is the average parasitemia of the negative control group and *B* is the average parasitemia of the test group.

#### 2.7.3. Prophylactic Activity on Residual Infection (Prophylactic Test)

Prophylactic activity was assessed according to the method described by Peters [19]. Mice were randomly divided into groups of six mice each and orally received BIWS-4b or physiological saline (negative control) for 4, 6, 8 or 10 consecutive days prior to infection. Twenty-four hours after the final administration of drug or saline, the mice were inoculated with *P. yoelii*. Seventy-two hours after inoculation, parasitemia levels were assessed in blood smears from tail blood of each mouse, and parasitemia (%) and average chemosuppression (%) were calculated.

### 2.8. Evaluation of Immunostimulating Activity In Vivo

#### 2.8.1. Drug Administration

The mice were randomly divided into groups of six mice each: a control group (administered physiological saline) and 100 and 200 mg kg^−1^ BIWS-4b groups. The drugs were given orally in doses of 0.2 ml for 4, 6, 8 or 10 consecutive days. Twenty-four hours after the final administration of drug or saline, mice in the malaria-bearing groups were inoculated with *P. yoelii* while the control group was used to assess the macrophage phagocytosis and lymphocyte proliferation activities of BIWS-4b in normal mice. Seventy-two hours after inoculation, the macrophage phagocytosis and lymphocyte proliferation activities of BIWS-4b in malaria-bearing mice were evaluated.

#### 2.8.2. Phagocytosis of Macrophage Assay

Chicken red blood cells (CRBC) were used to assess macrophage phagocytosis as described by Yang et al. [20]. Briefly, on the last day, 1 ml of 20% (v/v) CRBC was intraperitoneally injected into each mouse, and the mice were sacrificed 30 min later. Physiological saline (2 ml) was injected into the abdominal cavity, and then 1 ml fluid was collected to make a smear from each mouse. After incubating at 37°C for 30 min in a humidified 5% CO_2_ incubator, the smears were centrifuged to remove the supernatant, and the macrophages were fixed with methanol and stained by Giemsa-Wright for 7–10 min. The phagocytosis index was measured by counting the number of phagocytosed CRBC per every 100 macrophage cells, and the phagocytosis rate was measured by counting the number of macrophages containing CRBC per every 100 macrophage cells.

#### 2.8.3. Lymphocyte Proliferation Assay

Spleens were aseptically extirpated from the immunized mice. Spleen single cell suspensions were pooled in ice-cold Hank's solution by filtering through sieve mesh. Spleen cells were depleted of erythrocytes in Tris_2_NH_4_Cl (0.16 M, Tris_2_NH_4_Cl, pH 7.2), due to low-osmosis, followed by two washes in Hank's solution and resuspending in complete RPMI 1640 medium. Splenocyte activity was measured above 95% as assessed by the trypan blue dye exclusion method.

The lymphocyte proliferation assay was followed as described by Lee et al. [21] with slight modification. Briefly, spleen lymphocytes were seeded into 96-well flat-bottom microtiter plates at 5 ×10^6^ cells ml^−1^ and cultured in RPMI 1640 medium contained ConA (5.0 *μ*g ml^−1^) or LPS (10.0 *μ*g ml^−1^). The plates were incubated at 37°C in a humidified atmosphere with 5% CO_2_. After 44 h, 20 *μ*l of MTT solution (5 *μ*g ml^−1^) was added to each well and the cells were incubated for an additional 4 h. After aspirating the supernatant from the wells, 100 *μ*l of DMSO was added to dissolve formazan crystal. The absorbance at 570 nm was measured using a Bio-Rad microplate reader (Model 550, USA).

### 2.9. Statistical Analysis

All data were expressed as the mean ± SD of six replicates and examined for statistical significance with Student's *t*-tests. A result is considered statistically significant when *P* < .05. One-way ANOVA was conducted to assess significant differences among the treatments as a whole in order to avoid the error inherent in performing multiple *t*-tests.

## 3. Results

### 3.1. Isolation and Purification of BIWS-4b

Fruit bodies of *B. inquinans* (Fries) were treated with hot water, and extracted polysaccharides were precipitated in ethanol. Deproteination by the Sevag method gave an 8% yield of the polysaccharide (BIW) from dried fruit bodies. BIW was composed of glucose, mannose and galactose in a molar ratio of 1.0 : 1.3  : 1.0, respectively. A freeze-thaw cycle of the polysaccharide resulted in two fractions: BIWP (precipitate, 19%) and BIWS (supernatant, 63%). Analysis of BIWP revealed glucan, which was further purified by Sephadex G-75 gel filtration chromatography to give a homogeneous *β*-(1→6)-d-glucan [12].

BIWS consists of glucose, mannose and galactose in a molar ratio of 1 :  1 : 1 and exhibits a wide molecular weight distribution from 15 to 50 kDa. It was fractionated by gradient precipitation in 30, 50, 70 and 90% ethanol, which resulted in the fractions BIWS-1, BIWS-2, BIWS-3 and BIWS-4, respectively. BIWS-1, BIWS-2 and BIWS-3 showed wide distributions on Sepharose CL-6B or Sephadex G-75 elution profiles, while BIWS-4 gave two relatively narrow peaks on Sephadex G-75. Hence, BIWS-4 was fractionated on a Sephadex G-75 column (1.5  ×  100 cm) into two populations: BIWS-4a collected in the void elution volume (11% yield) and BIWS-4b (72% yield) collected in the effective fractionation range as a main portion. BIWS-4a is composed of glucose, mannose and galactose in a molar ratio of 5.7 : 1.0 : 8.0. Because of its low yield and wide molecular weight distribution, BIWS-4a was not studied further.

### 3.2. Analyses of BIWS-4b

**Figure 2:**
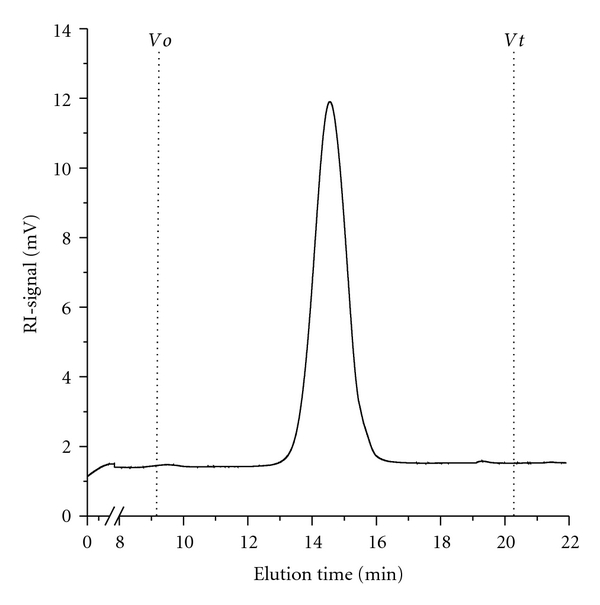
HPSEC elution profile of BIWS-4b.

BIWS-4b is a light brown powder and its total carbohydrate content was determined to be 96% by the phenol-H_2_SO_4_ method. The UV spectrum of BIWS-4b contained no peaks at 260 or 280 nm, and the Bradford assay was negative, which indicated that BIWS-4b did not contain protein and nucleic acid. A limulus amebocyte lysate (LAL) assay revealed less than 0.015 EU (endotoxin units) mg^−1^, which indicates a negative result. Analysis of sugar content showed that BIWS-4b consists of mannose (27.2%), glucose (15.5%) and galactose (57.3%). BIWS-4b gives a narrow symmetric peak by HPSEC (Figure 2), and its molecular weight was estimated to be 7.4 kDa (polydispersity index, Mw/Mn: 1.35), which suggests that BIWS-4b is a homogenous polysaccharide fraction. 

**Figure 3:**
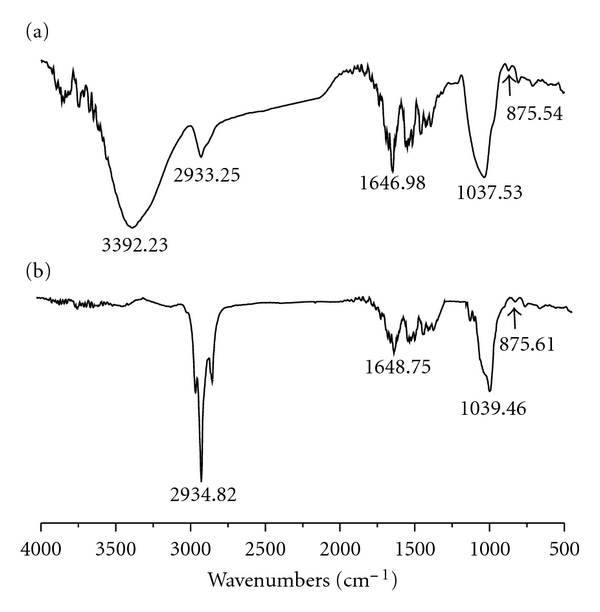
FT-IR spectra: (a) BIWS-4b and (b) partially methylated BIWS-4b.

**Figure 4:**
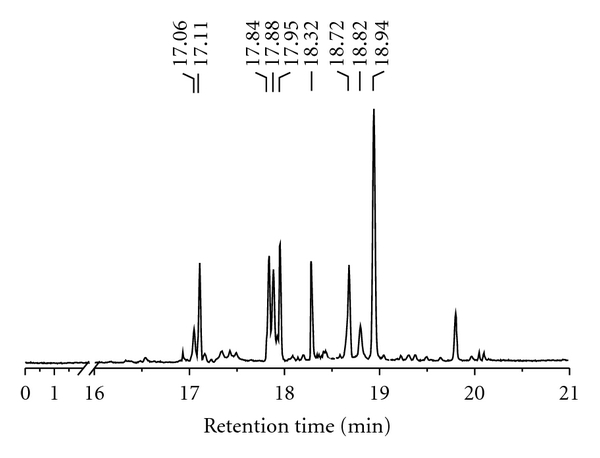
GC profile of BIWS-4b in methylation analysis.

The specific rotation measurement of BIWS-4b is [*α*]_D^20^_ –51.9° (*c* 0.2, H_2_O). The high negative rotation suggests the dominating presence of *β*-glycosidic linkages. The FT-IR spectrum of BIWS-4b (Figure 3) shows absorption of *β*-type glycosidic linkages at 875.5 cm^−1^. The other absorption bands at 3392.2 cm^−1^ (hydroxyl stretching vibration), 2933.3 cm^−1^ (C–H stretching vibration), and 1647.0 cm^−1^ (bound water) were from the corresponding sugar residues. Methylation products were sequentially acetylated and reduced to give seven methylated alditol acetates (Figure 4). This procedure revealed that galactosyl residues were (1→6)-linked, (1→5)-linked and (1→5,6)-linked Gal*f* units; glucosyl residues were (1→4)-linked and non-reducing terminal Glc*p* units; mannosyl residues were (1→2)-linked, (1→6)-linked, (1→2,6)-linked and non-reducing terminal Man*p* units. The ratios of each residue are listed in Table 1. 

**Figure 5:**
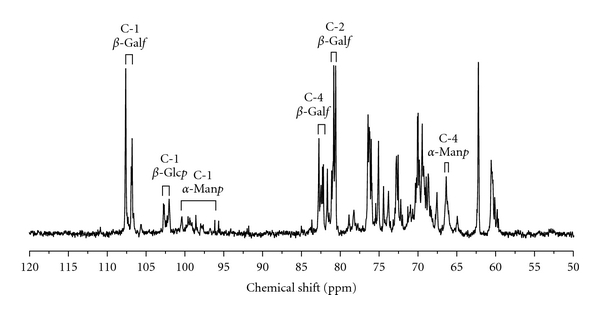
^13^C NMR spectrum of BIWS-4b. Solvent: D_2_O, temperature: 20°C.

The structural features of BIWS-4b were further analyzed by ^13^C NMR; assignments of carbon atom signals are listed in Table 2 and Figure 5. According to NMR data in the literatures [22–28], the anomeric carbon signals at *δ* 107.73, 107.03 and 106.92 can be attributed to C-1 of three different linkages of *β*-Gal*f* residues; *δ* 102.43 and 102.19 correspond to C-1 of *β*-Glc*p* and *δ* ~ 101.19 to 97.24 correspond to C-1 of *α*-Man*p*. These results were consistent with those of methylation analysis. Combining the results from methylation and NMR analyses, we deduced that BIWS-4b was mainly composed of (1→6)-linked, (1→5)-linked, and (1→5,6)-linked *β*-Gal*f* units, as well as (1→4)-linked, non-reducing terminal *β*-Glc*p* units, and (1→2)-linked, (1→6)-linked, (1→2,6)-linked and non-reducing terminal *α*-Man*p* units. 

### 3.3. Anti-Malarial Activity of BIWS-4b

#### 3.3.1. Anti-Malarial Activity of BIWS-4b on Early Infection

**Figure 6:**
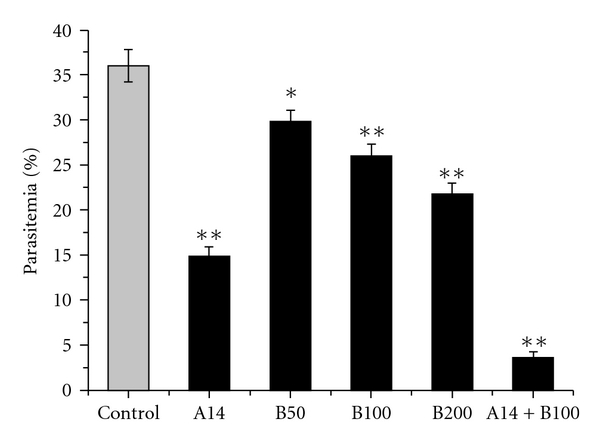
Anti-malarial effect of BIWS-4b on early infection. After inoculating with *P. yoelii*, the mice were orally treated with A: artesunate (14 mg kg^−1^), B: BIWS-4b (50, 100 and 200 mg kg^−1^), or combination of A and B for four consecutive days. Twenty-four hours after the final administration of drug or saline, parasitemia (%) was assessed in blood smears from tail blood of each mouse. Each value represents the mean ± S.D. The significant values are indicated by *P< .05 and **P < .01.

The anti-malarial activity of BIWS-4b was tested in mice by the 4-day method. As shown in Figure 6, parasitemia of BIWS-4b treated groups was significantly down (*P*<  .05 or *P*<  .01), in a dose-dependent manner, compared with the control group. Correspondingly, chemosuppression was significantly elevated. Chemosuppression was up 27.84% in mice administered oral doses of 100 mg kg^−1^ day^−1^ compared to controls. Artesunate is an effective anti-malarial drug used in the current market. Artesunate-induced chemosuppression was 58.59% at a dose of 14 mg kg^−1^ day^−1^. Co-administration of BIWS-4b and artesunate resulted in 89.85% chemosuppression, which is higher than the sum of the suppression caused by BIWS-4b and artesunate individually, indicating that BIWS-4b and artesunate acted synergistically. 

#### 3.3.2. Anti-Malarial Activity of BIWS-4b on Residual Infection

**Figure 7:**
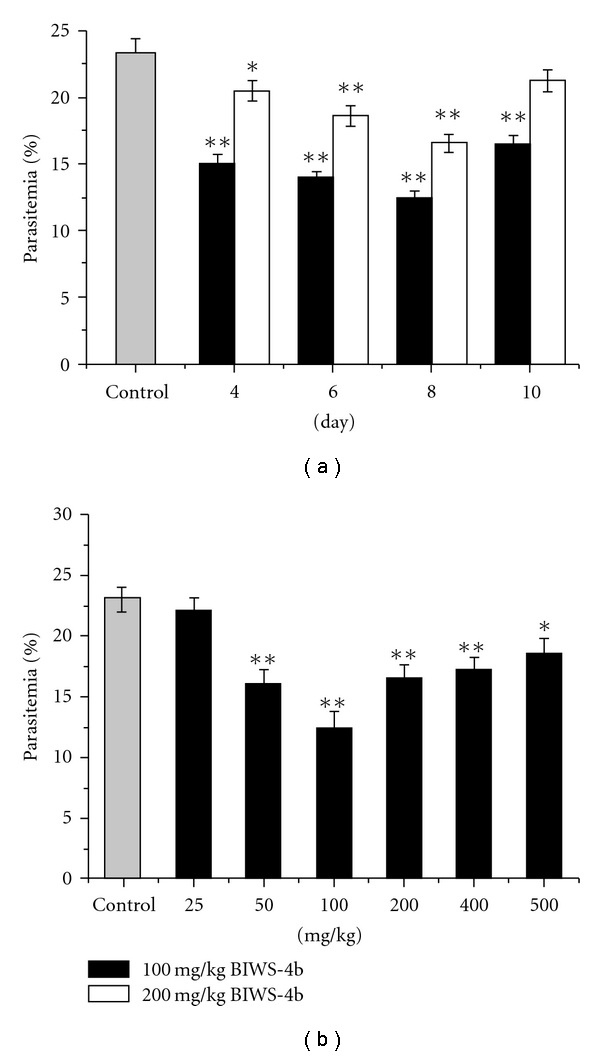
Anti-malarial effect of BIWS-4b on residual infection: (a) the mice were given BIWS-4b at 100 mg kg^−1^ and 200 mg kg^−1^ for 4, 6, 8, or 10 consecutive days; (b) the mice were given BIWS-4b at 25, 50, 100, 200, 400 and 500 mg kg^−1^ for 8 consecutive days. Twenty-four hours after the final administration of drug or saline, the mice were inoculated with *P. yoelii*. Seventy-two hours after inoculation, parasitemia (%) was assessed in blood smears from tail blood of each mouse. Each value represents the mean ± S.D. The significant values are indicated by *P < .05 and **P < .01.

The protective effect of BIWS-4b against malarial infection was examined by a residual infection assay in mice. The polysaccharides were administrated to mice followed by inoculation with *P. yoelii*. Our results indicate that BIWS-4b significantly decreased infection by *P. yoelii* (Figure 7(a)), which suggests that BIWS-4b enhanced the protective ability of mice against malaria. The protective ability increased with the time of administration, peaking on the eighth day. BIWS-4b significantly increased chemosuppression in the dose range of 25–500 mg kg^−1^ compared with the control group (*P*<.05 or *P*<.01) (Figure 7(b)); the data form a bell-shaped curve. At a dose of 100 mg kg^−1^, chemosuppression reached its highest level of 46.21% on the eighth day.

### 3.4. Immunological Activity of BIWS-4b

#### 3.4.1. Effect on Macrophage Phagocytosis

**Figure 8:**
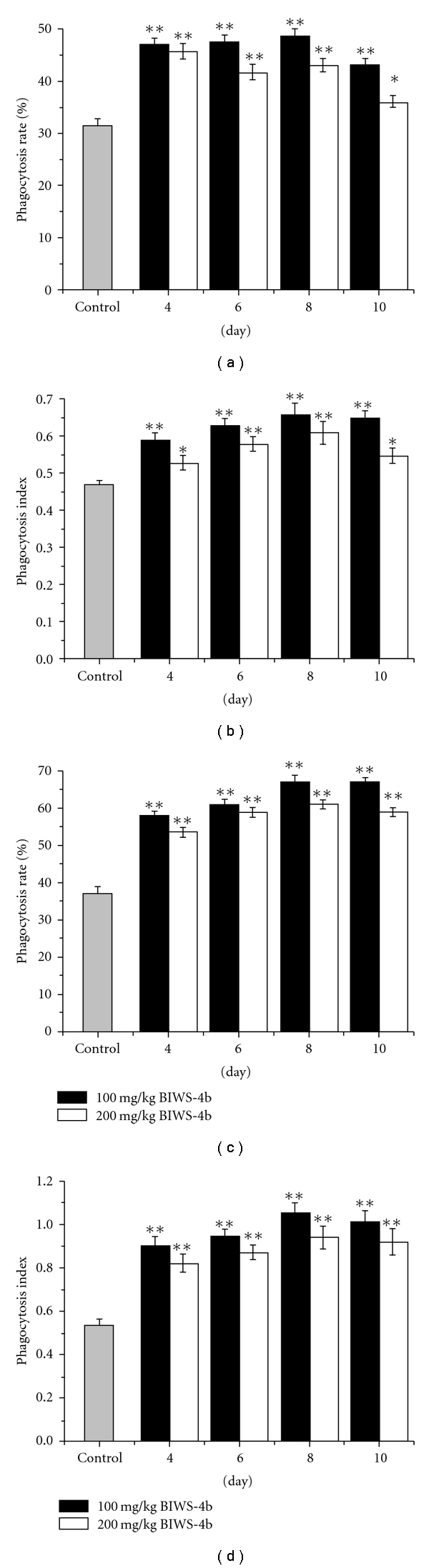
Effect of BIWS-4b on macrophage phagocytosis in normal mice and malaria-bearing mice: (a) phagocytosis rate in normal mice, (b) phagocytosis index in normal mice, (c) phagocytosis rate in malaria-bearing mice, (d) phagocytosis index in malaria-bearing mice. Each value represents the mean ± SD. Significant differences from the negative control were evaluated using student's *t*-test: *P <  .05, **P < .01.

The effect of BIWS-4b on macrophage phagocytosis was determined using chicken red blood cells (CRBC) in malaria-bearing mice and normal mice. As shown in Figure 8, the phagocytic rate and the phagocytic index were significantly increased compared with the control group by BIWS-4b treatment at doses of 100 and 200 mg kg^−1^ (*P*< .01). At a dose of 100 mg kg^−1^ BIWS-4b, macrophage phagocytosis was enhanced to a greater extent than at a dose of 200 mg kg^−1^, and the former dose caused the most macrophage phagocytosis in malaria-bearing mice and normal mice at the eighth day. These results are consistent with residual infection. 

#### 3.4.2. Effect on Lymphocyte Proliferation

**Figure 9:**
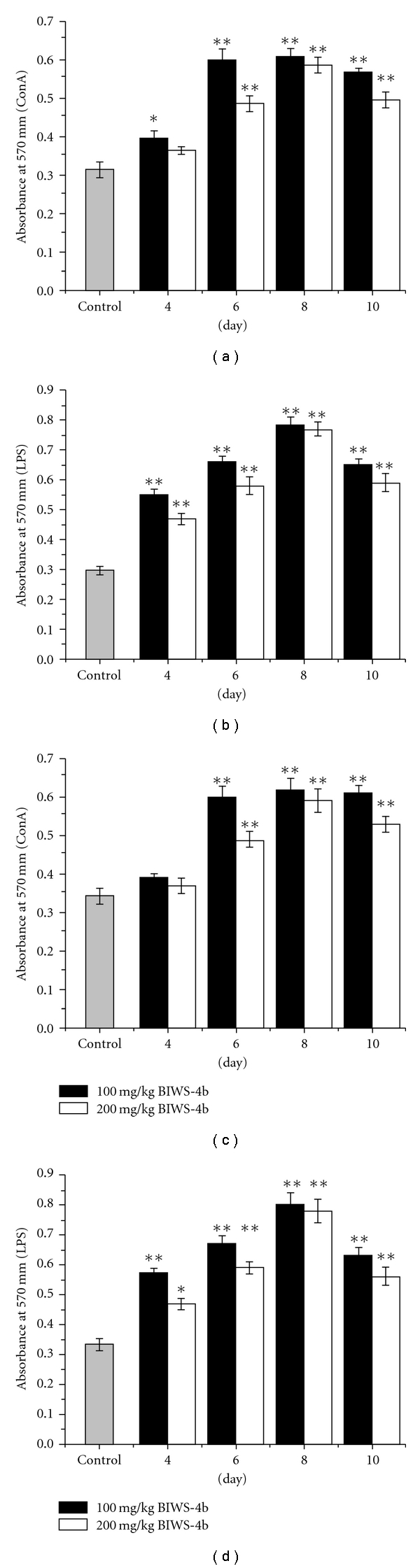
Effect of BIWS-4b on lymphocyte proliferation in normal mice and malaria-bearing mice: (a) ConA-induced T-lymphocyte proliferation in normal mice, (b) LPS-induced B-lymphocyte proliferation in normal mice; (c) ConA-induced T-lymphocyte proliferation in malaria-bearing mice; (d) LPS-induced B-lymphocyte proliferation in malaria-bearing mice. Each value represents the mean ± SD. Significant differences from the negative control were evaluated using Student's *t*-test: *P < .05, **P < .01.

Splenic lymphocyte proliferation is usually followed to evaluate a general effect on immune cells. Lymphocyte proliferation induced by ConA reflects T-lymphocyte activity while proliferation induced by LPS reflects B-lymphocyte activity. The effects of BIWS-4b on ConA- and LPS-induced lymphocyte proliferation were tested *in vivo* by the MTT method. As shown in Figure 9, in the presence of ConA or LPS, BIWS-4b significantly increased lymphocyte proliferation in malaria-bearing or normal mice compared to the control group (*P* < .05). These results indicate that BIWS-4b was able to activate both T and B cells. At a dose of 100 mg kg^−1^, BIWS-4b induced higher effect on lymphocyte proliferation than that at a dose of 200 mg kg^−1^ and, similar to its effect on macrophage phagocytosis, lymphocyte proliferation both in malaria-bearing mice and normal mice peaked at the eighth day. Taken together, the data suggest that BIWS-4b can stimulate T/B-cells to suppress the spread of malaria. 

## 4. Discussion

Polysaccharides from fungi often possess immunological activities that can protect the body from microbial and parasitic attack [29–31]. Although some polysaccharides from ascomycetous lichens are reported to be composed of galactofuranose, glucopyranose and mannopyranose residues [25, 27, 28, 32–37], little work about possible anti-malarial activity has been performed. Lentinan (1,3-*β*-d-glucan) has recently been investigated for its anti-malarial activity using the *P. yoelii* blood-stage infection model and found to possess prophylactic potential for the treatment of malaria *via* immunostimulation [9]. In this article, we isolated, for the first time, a heteropolysaccharide fraction (BIWS-4b) containing *α*-d-Man*p*, *β*-d-Glc*p* and *β*-d-Gal*f* from the non-lichenized edible mushroom *B. inquinans* (Fries). Considering the complex structure of BIWS-4b, we supposed it may have potential biological activities. Therefore, BIWS-4b was evaluated for its complementary therapy and prophylactic activities against malaria as well as its immunostimulating activity. The results indicated that BIWS-4b had markedly suppressive activity on early or residual infection. Thus, it is possible that BIWS-4b could be used as a complementary drug or protective agent against malaria.

Artesunate is an effective drug currently used to cure malarial infection. The endoperoxide moiety of its molecular structure can oxidize parasitic membranes and react with heme to form a cytotoxic carbon-centered radical intermediate that further reacts with susceptible groups within parasite enzymes and lipids [38–40]. However, artesunate has some negative side effects including interfering with some aspects of the host immune system, resulting in the difficulty of establishing a long-lasting protective immunity against malaria [41]. Therefore, artesunate-based combination therapy was recommended by the World Health Organization (WHO) as a new prospective paradigm [42]. Lymphocytes are the key effecter cells of the mammalian immune system and stimulating macrophages is another way to enhance immunological activity [43, 44]. BIWS-4b significantly enhanced spleen lymphocyte proliferation upon stimulation by ConA or LPS and augmented macrophage phagocytosis in malarial-bearing or normal mice. In our experiments, it was observed that both immunological enhancement of BIWS-4b and its protection against malarial infection peaked at 100 mg kg^−1^ on Day 8, which implied that its anti-malarial activity might be intermediated by enhancing host immune competence. In combination with artesunate, BIWS-4b was able to recover immune system functionality that was previously suppressed by artesunate in mice. The synergistic effect of BIWS-4b and artesunate may be utilized to enhance and restore artesunate-suppressed immune function. Similar results were observed for lentinan [9].

The prophylactic activity against malaria and the immunological enhancement of BIWS-4b exhibited a bell-shaped curve. One interpretation of this effect is that signal transduction mechanisms underlying the action of lymphocytes and macrophages might be regulated downward by exposure to a higher dosage of BIWS-4b [45]. An alternative interpretation is that BIWS-4b has not only effects which promote the immune system, but also those which inhibit their system, and the promoting mechanisms might be masked by its inhibitory effects at a higher dosage [46]. Similar results have been observed in some polysaccharides when used as complementary drugs for anti-cancer treatments [47–50], such as the tumor growth inhibition of a *Cordyceps sinensis* polysaccharide [47] and the TNF-*α* level increase by an *Aloe vera* polysaccharide [48].

**Figure 10:**
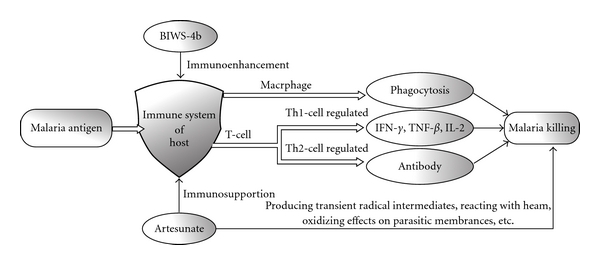
The deduced anti-malarial action of BIWS-4b: when infected with malaria, the host generates a protective immune response, including cellular and humoral immunity. BIWS-4b can significantly enhance the immune competence of the host and exhibits remarkable prophylactic activity against malaria. In combination with artesunate, BIWS-4b can restore the artesunate-suppressed immune function and enhance the anti-malarial activity of artesunate.

In conclusion, a water-soluble heteropolysaccharide BIWS-4b was purified from fruit bodies of *B. inquinans* (Fries). It was found to contain (1→6)-, (1→5)- and (1→5,6)-linked *β*-Gal*f* units, as well as (1→4)-linked non-reducing terminal *β*-Glc*p* units, and (1→2)-linked, (1→6)-linked, (1→2,6)-linked and non-reducing terminal *α*-Man*p* units. BIWS-4b was shown to possess marked suppressive activity against early infection of malaria by the 4-day method in mice. Residual infection and immunological assays indicated that BIWS-4b had significant prophylactic activity against malaria by stimulating the immune system. Moreover, BIWS-4b increased anti-malarial activity of artesunate at early infection by oral administration in combination. The deduced anti-malarial action of BIWS-4b was shown in Figure 10. Therefore, BIWS-4b could be considered a potential adjuvant of anti-malarial drugs. 

## Funding

National Natural Science Foundation of China (no. 30570417, 30770489) and the Natural Science Foundation of Jilin Province (no. 20070710).

## Figures and Tables

**Table 1:** Methylation analysis of BIWS-4b.

Partially methylated glycitol	Molar ratio^a^	Retention time (min)	Linkage type
1,4,5-Tri-acetyl-2,3,6-tri-*O*-methyl galactitol	4.6	17.95	→5)-Gal*f*-(1→
1,4,5,6-Tetra-acetyl-2,3-di-*O*-methyl galactitol	3.1	17.88	→5,6)-Gal*f*-(1→
1,4,6-Tri-acetyl-2,3,5-tri-*O*-methyl galactitol	7.6	18.94	→6)-Gal*f*-(1→
1,4,5-Tri-acetyl-2,3,6-tri-*O*-methyl glucitol	2.2	18.32	→4)-Glc*p*-(1→
1,5-Di-acetyl-2,3,4,6-tetra-*O*-methyl glucitol	2.6	17.11	Glc*p*-(1→
1,2,5-Tri-acetyl-3,4,6-tri-*O*-methyl mannitol	2.4	17.84	→2)-Man*p*-(1→
1,5-Tri-acetyl-2,3,4,6-tri-*O*-methyl mannitol	1.0	17.06	Man*p*-(1→
1,2,5,6-Tetra-acetyl-3,4-di-*O*-methyl mannitol	1.2	18.82	→2,6)-Man*p*-(1→
1,5,6-Tri-acetyl-2,3,4-tri-*O*-methyl mannitol	2.9	18.72	→6)-Man*p*-(1→

^a^Relative molar ratio, calculated from the ratio of peak area.

**Table 2:** ^13^C NMR chemical shifts of BIWS-4b in D_2_O.

Residue	*δ* (ppm)					
	C-1	C-2	C-3	C-4	C-5	C-6
→5)-*β*-Gal*f*-(1→	107.73	80.93	76.81	81.18	75.47	62.73
→5,6)-*β*-Gal*f*-(1→	107.03	81.45	76.40	82.50	74.20	68.01
→6)-*β*-Gal*f*-(1→	106.92	82.00	76.65	83.98	69.89	69.70
→4)-*β*-Glc*p*-(1→	102.43	73.18	74.30	79.09	74.83	60.64
*β*-Glc*p*-(1→	102.19	72.96	74.20	69.12	75.83	60.32
*α*-Man*p*-(1→	101.19	69.12	69.54	66.56	72.40	60.10
→2)-*α*-Man*p*-(1→	99.45	77.25	68.76	66.67	72.40	60.10
→6)-*α*-Man*p*-(1→	98.37	69.12	69.54	66.58	71.95	64.89
→2,6)-*α*-Man*p*-(1→	97.24	77.62	69.40	66.67	71.95	64.92
